# Malawi ICEMR Malaria Research: Interactions and Results Influencing Health Policies and Practices

**DOI:** 10.4269/ajtmh.21-1265

**Published:** 2022-10-13

**Authors:** Charles Mangani, Themba Mzilahowa, Lauren Cohee, Michael Kayange, Peter Ntenda, Alick Sixpence, Austin Gumbo, Sosten Lankhulani, Jessy Goupeyou-Youmsi, Edward Walker, Miriam Laufer, Clarissa Valim, Karl Seydel, Mark L. Wilson, Terrie Taylor, Don P. Mathanga

**Affiliations:** ^1^ Department of Epidemiology and Biostatistics, School of Public Health, Kamuzu University of Health Sciences, Blantyre, Malawi;; ^2^ Malaria Alert Center, Kamuzu University of Health Sciences, Blantyre, Malawi;; ^3^ Department of Pediatrics, Center for Vaccine Development and Global Health, University of Maryland School of Medicine, Baltimore, Maryland;; ^4^ National Malaria Control Program, Ministry of Health, Lilongwe, Malawi;; ^5^ Department of Microbiology and Molecular Genetics, Michigan State University, East Lansing, Michigan;; ^6^ Department of Global Health, Boston University School of Public Health, Boston, Massachusetts;; ^7^ Department of Osteopathic Medical Specialties, College of Osteopathic Medicine, Michigan State University, East Lansing, Michigan;; ^8^ Department of Epidemiology, School of Public Health, University of Michigan, Ann Arbor, Michigan

## Abstract

Malaria remains a threat to public health in Malawi. It is well acknowledged that malaria research and robust evidence can have an impact on malaria policy and practice, resulting in positive population health gains. We report policy-relevant research contributions that the Malawi International Center of Excellence for Malaria Research (ICEMR) in partnership with local and international collaborators has made. Findings from our ICEMR studies have shown that long-lasting insecticide-treated bed nets (LLINs) impregnated with piperonyl butoxide reduced mosquito blood feeding more compared with conventional LLINs. On the other hand, we showed that few LLINs are maintained up to the end of their 3-year life span, and that older nets are less effective. These results support the policy change decisions by the Malawi National Malaria Control Program to switch from conventional LLINs to piperonyl butoxide LLINs, and to conduct mass LLIN distribution campaigns every 2 years. Our studies on epidemiological patterns of malaria infection showed that school-age children have higher malaria infection rates and lower use of control measures compared with younger children and adults. These findings added to the evidence base that influenced the National Malaria Control Program to endorse school-based malaria interventions as part of its national policy. Research supported by the Malawi ICEMR is contributing to in-country policy decisions and to the implementation of evidence-based interventions. Through our long-term studies we intend to continue providing practical and policy-relevant evidence necessary, ultimately, to eliminate malaria infection in Malawi.

## INTRODUCTION

**Figure 1.**
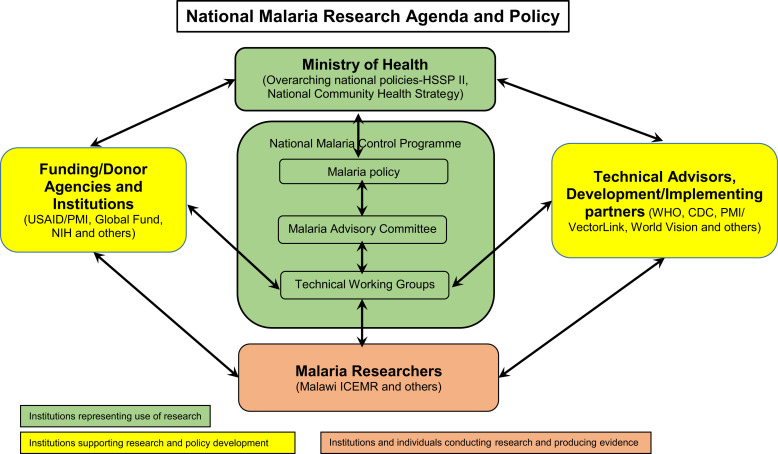
Malaria policy development context in Malawi. CDC = Centers for Disease Control and Prevention; HSSP II = Health Sector Strategic Plan II; ICEMR = International Center for Excellence in Malaria Research; NIH = National Institutes of Health; PMI = President’s Malaria Initiative, United States Agency for International Development; WHO = World Health Organization. Adapted from Mwendela et al.^2^

Malaria continues to be a major public health issue in Malawi, with an estimated 4.4 million cases reported in 2020.^1^ Malawi’s entire population of 19 million is at risk, and transmission occurs throughout the year. The Malawi National Malaria Control Program (NMCP) has the overall responsibility for developing malaria policies and strategies, and for providing technical leadership to the Ministry of Health and its partners. Key among the partners are funding organizations such as Global Fund, United States Agency for International Development/President’s Malaria Initiative (PMI), and academic and research institutions such as the Malaria Alert Center (MAC) (Figure 1,^2^). Traditionally, the Malawi NMCP has used evidence from malaria research to direct policy.^3^ For example, in 1993, based on research evidence, Malawi was the first country to switch from chloroquine to sulfadoxine–pyrimethamine as a first-line antimalaria drug treatment of uncomplicated malaria.^4^ Implementation of other evidence-based strategies and policies has also helped to reduce *Plasmodium* infection prevalence in children younger than 5 years by nearly 50% between 2010 and 2017.^5^ However, the decline in malaria morbidity in Malawi has not decreased at the pace seen in surrounding countries. Disease burden still remains high. In 2020, annual malaria illness incidence varied by district from ∼155 to ∼1,050 cases per 1,000 population.^6^

The long-term vision of Malawi’s NMCP is to eliminate malaria disease, with the intermediate goal of reducing the burden of malaria to a level where it is no longer a disease of public health significance.^7^ The 2017–2022 Malaria Control Strategic Plan prioritizes reducing malaria morbidity and mortality through 1) vector control, primarily by the distribution and use of quality long-lasting insecticide-treated bed nets (LLINs) and localized use of indoor residual spraying (IRS); 2) prompt access to effective treatment; and 3) widespread use of intermittent preventive treatment in pregnancy.^7^^,^^8^ The success of the strategic plan depends on continued generation of local research findings to inform evidence-based policy development.^7^ The Malawi International Center of Excellence for Malaria Research (ICEMR) has, for the past decade, conducted malaria policy-relevant research. This report highlights evidence generated by the Malawi ICEMR, through interactions with Malawi governmental agencies and national and international partners, and the potential impact of this research output on influencing malaria policy and practice.

## MALAWI ICEMR: OVERVIEW, COLLABORATION, AND PARTNERSHIPS

In 2010, the U.S. National Institute of Allergy and Infectious Diseases established the ICEMR program. The aim was to address existing deficiencies in malaria research in endemic settings; most research projects were observed to have a narrow scientific scope, done in single field sites with a limited catchment area, and a population at risk that was relatively homogenous.^9^^,^^10^ To address these gaps, the ICEMR program was designed to conduct multidisciplinary research on malaria in different endemic eco-epidemiological settings. The first ICEMR program ran from 2010 to 2017 in 10 centers, including Malawi. The first Malawi ICEMR program (ICEMR 1) aimed at understanding burden and patterns of malaria infection and disease, and analyzing transmission by *Anopheles* vector species in both urban and rural ecological settings. The program was renewed in 2017 for another 7 years. The overall goal of the second Malawi ICEMR (ICEMR 2) is to identify why the standard malaria control and prevention efforts in Malawi have had only a limited impact on malaria disease incidence and parasite prevalence. Ongoing studies in ICEMR 2 aim at understanding factors that influence effectiveness of LLINs, as well as vector and human behavioral risk factors for *Plasmodium* infection transmission and malaria disease. Both ICEMR projects have been supported by the Malawi NMCP, with a view of using data generated for the development of new policies and practices.

The Malawi ICEMR program is implemented through the MAC, a research center established in 2001 as part of Kamuzu University of Health Sciences (formerly University of Malawi, College of Medicine). It focuses on operational and implementation research studies on malaria, including entomological monitoring. In addition to the Malawi ICEMR, the MAC collaborates with the United States Agency for International Development/PMI, the CDC, the WHO, World Vision International, VectorLink Malawi, Save the Children, and universities to support local and global agencies with data that can be used to drive malaria policies (Figure 1,^2^).

In Malawi, malaria policy decisions are initiated in various technical working groups (TWGs) comprised of experts from academia, key implementation partners such as non-governmental organizations, and the donor community. In Malawi, there are four main TWGs: Malaria Integrated Vector Management, Malaria Case Management, Intermittent Preventive Treatment in Pregnancy, and Monitoring and Evaluation. Their mandate is to review new evidence, evaluate global trends and guidelines, and advise the NMCP on new policy initiatives and how to address implementation bottlenecks. The local malaria research-to-policy framework is used; local evidence is prioritized in policy decision making.^2^^,^^4^^,^^11^ Recommendations from the TWGs are referred to the Malaria Advisory Committee (Figure 1,^2^), which is the ultimate body that advises the NMCP on policy changes. Malawi ICEMR scientists are members of the TWGs, and some sit on the Malaria Advisory Committee. Their involvement in these key policy decision-making bodies means they are able to leverage data from all research projects on which they are collaborating as they participate in the development of national malaria policies, and at the same time gain an understanding of the key research needs of the policymakers.

## VECTOR CONTROL

The use of LLINs is the mainstay of malaria vector control in Malawi. Since 2012, the Malawi NMCP has conducted nationwide mass LLIN distribution campaigns every 3 years, with the goal of increasing LLIN coverage and use.^12^ As a result of these efforts, household access to a LLIN increased from 37% in 2012 to 63% in 2017, with a corresponding increase in LLIN use, from 41% to 55%.^12^ In addition, IRS has been deployed, although at a smaller scale. Recent efforts in IRS implementation started in 2007 with one district, and were scaled-up to seven districts in 2012. Because of inadequate funding, IRS is being conducted in only four of the country’s 29 districts. Despite enhanced vector control activities, data suggest that the decline in malaria incidence has recently stalled in Malawi, as in some malaria-endemic countries.^1^ The decreasing effectiveness of LLINs may be contributing to this plateau, and for this reason, monitoring for effectiveness of both—LLINs and IRS—is imperative.

### Monitoring the effectiveness of LLINs in malaria control in Malawi.

Researchers at the MAC in Malawi have been monitoring the effectiveness of LLINs for malaria control in Malawi since 2013, comparing incidence of *Plasmodium* infection in LLIN users and non-users in areas of Malawi where pyrethroid resistance in the primary vector (*Anopheles funestus*) was high. In two CDC-funded studies, use of LLINs was associated with a 30% reduction in the incidence of malaria infection compared with non-users in one study,^13^ despite high levels of pyrethroid resistance and no difference in protection detected in a second study.^14^ Based on these findings and similar evidence from other malaria-endemic countries,^15^ conventional pyrethroid-based LLINs remained the cornerstone of malaria control in Malawi until 2019, when more compelling evidence from epidemiological data^16^ showed that conventional LLINs were less effective than piperonyl butoxide (PBO)–LLINs. Therefore, the NMCP made a decision to move away from conventional LLINs completely. The Malawi ICEMR 2 program has expanded on objectives of these two foundational studies. These studies are designed to identify factors impinging on the effectiveness of LLINs over their life span and to compare the effect of conventional LLINs and PBO-LLINs on malaria indicators. We have established community-based cohort and cross-sectional qualitative studies to identify and understand barriers to LLIN program success. However, the COVID-19 pandemic prompted us to shut down cohort follow-up 2 months earlier than planned and to halt temporarily other research activities. Analysis of the findings of these ICEMR 2 studies is forthcoming.

In support of the Malawi government’s nationwide LLIN distribution campaigns, the MAC had previously carried out analyses of insecticide resistance in vector populations.^17^ Results revealed a spectrum of phenotypic and genotypic resistance attributes, and in particular showed that many populations of *An. funestus *and* Anopheles arabiensis* are resistant to pyrethroids in Malawi. Furthermore, experimental huts studies funded by the PMI/VectorLink of PermaNet 3.0 and Olyset Plus showed that PBO-enhanced LLINs performed better than the conventional nets in reducing mosquito blood feeding^18^ in Malawi. In a countrywide facility-based study by Topazian et al.,^16^ malaria incidence during the high transmission months declined by 60.8%, from 28.6 cases per 100 people in 2018 to 11.2 cases per 100 people in 2019 in five districts that received PBO-LLINs compared 23.5% (26.8 cases per 100 people in 2018 to 20.5 cases per 100 people in 2019) in districts with conventional LLINs. These findings and WHO recommendations on deployment of PBO-LLINs^19^ formed the basis for the Malawi NMCP’s decision to switch from conventional nets to PBO-LLINs. Therefore, only PBO-LLINs will be distributed during the nationwide mass distribution campaigns and through routine distribution to pregnant women and children younger than 5 years.

For the past 3 years, the Malawi ICEMR 2 program has been evaluating the effectiveness of PBO-LLINs in two districts through community-based cohort studies, one with PBO-LLINs and the other with conventional LLINs. Results from our preliminary entomological study showed high rates of human feeding by vectors in both PBO-LLIN and conventional LLIN sites. However, the number of infectious *Anopheles* bites per person-year was 44 in the district deploying conventional LLINs and 22 in the district deploying PBO-LLINs. The human blood index was also significantly greater in in the district deploying conventional LLINs (0.96) than in the district deploying PBO-LLINs (0.89).^20^ Our preliminary work also shows that deployment of PBO-LLINs may have reduced mosquito–human contact because the human blood index was significantly less at the site where people used the PBO-LLINs. We are continuing to explore how effective PBO-LLINs will be in reducing *Plasmodium* transmission, especially over the 3-year life span of the LLINs.

### Physical durability of LLINs.

Measuring the physical condition of LLINs under field conditions is important for malaria control programs to guide decisions on how frequently to replace LLINs. Our team has shown that older LLINs are associated with greater rates of *Plasmodium falciparum* infection.^21^ It has also been shown that most of the nets had acquired holes through wear-and-tear by 2 years, and that there is net attrition of 20% or more within the first year of receiving LLINs (Mzilahowa, personal communication).These findings corroborate observations from other sub-Saharan African countries.^22^^,^^23^ The totality of these research findings has led to a major policy shift, in which the Malawi NMCP will now conduct mass distribution campaigns every 2 years starting in 2021, instead of every 3 years as in previous campaigns. The COVID-19 pandemic, however, caused a disruption in the distribution of LLINs and the nets were not deployed in some districts of the country. The national distribution campaign will be completed in May 2022. Ongoing studies in the Malawi ICEMR 2 program are continuing to explore the effect of net condition on malaria infection and disease.

## EPIDEMIOLOGY OF MALARIA IN MALAWI

### Malaria in school-age children.

The Malawi ICEMR^24^ corroborated earlier reports identifying high prevalence rates of malaria infection in school-age children.^25^ School-age children have also been identified as major contributors to transmission.^26^^–^^28^ Therefore, understanding the special role these children play in maintaining malaria is critical to moving toward the policy goal of transitioning from malaria control to malaria elimination.

Malawi ICEMR research,^29^ and recent studies from elsewhere,^30^^,^^31^ have shown that current malaria control interventions have not affected infections in school-age children effectively. Indeed, our investigations have demonstrated that these children are less likely than other age groups to use LLINs or to seek treatment in the formal health sector.^32^^,^^33^ Furthermore, infections in school-age children contribute disproportionately to perpetuating *P. falciparum* transmission.^27^ These studies have contributed to an important policy recommendation: interventions that specifically target schoolchildren that are likely to have larger, community-wide impacts that reduce malaria prevalence.^34^ In addition, the insight that school-age children are less likely to seek treatment from the formal sector has led the Malawi NMCP to endorse pilot programs of school-based testing and treatment.^35^^,^^36^ Furthermore, the NMCP included school-based LLIN distribution in the Malawi Malaria Strategic Plan (2015–2022), which is aimed at increasing access and use of LLINs.^8^ The Malawi ICEMR has also been conducting studies to identify the barriers to consistent use of malaria interventions, including LLINs among school-age children. Our preliminary findings have supported the design and funding of a clinical trial of school-based preventive treatment and feasibility studies of integrating school-based malaria interventions with ongoing and new school health programs, such as deworming programs.^37^ Last, Malawi ICEMR findings are being used to advocate for chemoprevention in school-age children as well as to develop integrated school health packages.^34^

### Malaria in urban settings.

Although the entire sub-Saharan African region is exposed to malaria risk, considerable heterogeneity in transmission patterns exists and is influenced by demographic, socioeconomic, and urban-versus-rural contexts. In general, it has been considered that people living in urban and peri-urban areas have a lower risk of malaria than those in rural settings.^38^ However, urban areas, even within the same country, are highly heterogeneous. Urbanization, which includes the colonization of land, changing population densities, evolution of infrastructure (including housing), and small-scale crop production, is heterogeneous with respect to malaria resulting in diverse ecologies, exposure patterns, and prevention opportunities.^39^

Our team has demonstrated that transmission occurs regularly in urban settings through the presence of *An. funestus* s.s and *An. gambiae *s.l. in the periphery of Blantyre city in Malawi.^40^ Furthermore, small-scale agriculture, poverty, and poor-quality housing are major influences of the local abundance of malaria vectors in urban settings.^40^ Our ICEMR data also show that, among residents of urban areas, travel to rural areas strongly increased risk of malaria illness.^41^ Thus, interventions targeting mobile urban dwellers may lead to reductions in malaria prevalence in urban settings. Indeed, we recently demonstrated how the urban–rural continuum of malaria risk poses challenges for intervention policies that ignore local conditions within the broader settings of “urban” and “rural.”^42^ Malawi currently does not have a stratified malaria control program because it is assumed that the entire population is at high risk of infection. However, research, including that from Malawi ICEMR, has reignited debate on whether a stratified malaria program that uses different combinations of interventions should be introduced in the country. In addition, currently there are no global or local policies developed specifically to address the challenges of urban malaria. Nevertheless, our findings are being used by the WHO to support the development of urban malaria policies (Mathanga, personal communication). Our findings could also guide future implementation and cost–benefit analysis studies evaluating optimal delivery of control measures that include a mix of those in current common use (e.g., LLINs, prompt diagnosis and treatment) and those currently used infrequently (IRS, larval control, and environmental management). The goal is to guide future policy development for urban dwellers with insufficient protection from malaria parasite transmission resulting from poor housing and living conditions, and limited financial resources.

## INTRODUCTION OF THE RTS, S VACCINE

Malawi is one of three countries implementing an RTS, S/ASO1 malaria vaccine pilot program. The aim is to evaluate the feasibility of delivering the vaccine within the Expanded Program on Immunization, investigate the safety signals identified during the phase 3 trials, and document the impact of the vaccine on mortality.^43^ Some of our ICEMR study sites are receiving the vaccine, whereas others are not. The NMCP also introduced IRS and PBO-LLINs in some of these same sites. Thus, the Malawi ICEMR is partnering with the NMCP in a unique opportunity to evaluate the interactions and impact of these interventions on malaria parasite transmission. Our preliminary data show that the odds of malaria infection were less in children who had received RTS, S or who used PBO-LLINs compared with those who had used conventional LLINs only. Our findings so far support the WHO recommendation for the broad use of the vaccine among young children in Africa and in other areas with moderate to high transmission of *Plasmodium falciparum*. Findings from our studies, in collaboration with the NMCP and the WHO, should have implications for policymakers, because understanding both the direct and indirect population-level benefits of malaria vaccination and PBO-LLINs will be critical when countries decide whether to adopt these intensive interventions. Our findings can also be used to enhance program effectiveness, both at national and international levels.

## CONCLUSION

The NMCP sits at the heart of malaria prevention and control efforts in malaria-endemic countries. Timely and high-quality research results are necessary to guide evidence-based policy and programmatic decision making and action effectively. The Malawi ICEMR was designed to help address knowledge gaps in malaria epidemiology and parasite transmission, to assess the use and impact of current malaria control strategies, and to guide the implementation of established and new interventions by providing evidence-based recommendations to policymakers. This article highlights policy-relevant research findings from the Malawi ICEMR that can influence malaria policy, both in Malawi and internationally. Evidence from our studies has strengthened malaria vector surveillance and insecticide resistance monitoring, inspired pilot programs for school-based malaria interventions, is contributing to the development of WHO policy on urban malaria, and is expanding our understanding of how the RTS, S malaria vaccine reduces disease risk. Continued and improved interaction between ICEMR researchers and policymakers is therefore central to promoting the translation of research into decision making and policy development, and also to advocating for more policy-relevant studies.

There are still outstanding policy questions that Malawi needs to answer, and these include the effectiveness of PBO-LLINs over their life span, effectiveness of IRS in reducing *Plasmodium* infection over the yearly spraying cycle, and appropriate malaria intervention mix and delivery strategies for school-age children, among others. Therefore, there is a stronger need for research funding agencies such as the NIH to continue investing in malaria research in Malawi to generate evidence that will guide policy and contribute to the Malawi NMCP goal of malaria elimination by 2030.
